# De Novo Detrusor Underactivity and Other Urodynamic Findings after Radical Prostatectomy: A Systematic Review

**DOI:** 10.3390/medicina58030381

**Published:** 2022-03-04

**Authors:** Maciej Oszczudłowski, Konrad Bilski, Mieszko Kozikowski, Jakub Dobruch

**Affiliations:** Urology Clinic, Centre of Postgraduate Medical Education, 01-813 Warsaw, Poland; konradbilski@gmail.com (K.B.); mieszkokozikowski9@gmail.com (M.K.); jdobruch@cmkp.edu.pl (J.D.)

**Keywords:** radical prostatectomy, detrusor underactivity, urodynamic study, detrusor overactivity, bladder outlet obstruction, impaired bladder compliance

## Abstract

*Background and objectives:* The aim of this systematic review is to evaluate the impact of radical prostatectomy (RP) on bladder function, with special attention towards detrusor underactivity investigated with the means of urodynamic evaluation. *Materials and Methods:* The review was performed in accordance with the PRISMA statement and was registered in the PROSPERO (ID#: CRD42020223480). The studied population was limited to men with prostate cancer who underwent urodynamic study prior to and after radical prostatectomy. Eight hundred twenty-seven studies were screened, with twenty-five finally included. A qualitative analysis was performed. Rates of detrusor underactivity (DU) before surgery were reported in eight studies and ranged from 1.6% to 75% (median of 40.8%). DU occurred de novo after RP in 9.1% to 37% of patients (median of 29.1%). On the other hand, preexisting DU resolved in 7% to 35.5% of affected men. Detrusor overactivity (DO) was the most frequently reported outcome, being assessed in 23 studies. The rate of DO preoperatively was from 5% to 76% (median of 25%). De novo was reported in 2.3–54.4% of patients (median of 15%) and resolved after RP in 19.6% to 87.5% (median of 33%) of affected patients. Baseline rates of bladder outlet obstruction (BOO) varied between studies from 19% to 59.3%, with a median of 27.8%. The most pronounced change after surgery was the resolution of BOO in 88% to 93.8% (median of 92%) of affected patients. *Results:* Rates of de novo impaired bladder compliance (IBC) varied from 3.2% to 41.3% (median of 13.3%), whereas the resolution of IBC was reported with rates ranging from 0% to 47% (median of 4.8%). *Conclusions:* BOO, DO, and DU are frequently diagnosed in men scheduled for RP. BOO is improved after RP in most patients; however, there is still a substantial rate of patients with de novo DU as well as DO which may impair functional outcomes and quality of life.

## 1. Introduction

Prostate cancer (PCa) is the second most common cancer in men worldwide, accounting for 15% of all diagnosed cancers [1]. Implementation of prostate-specific antigen testing and the extended life span of elderly men in developed countries led to a substantial increase in PCa incidence, followed by rising numbers of younger patients with long life expectancy subjected to radical prostatectomy (RP). Moreover, novel tools based on genetic alterations and epigenetic interactions are evaluated for the determination of indolent or aggressive tumour behaviour [2]. This phenomenon resulted in a growing interest in novel surgical techniques that would limit the functional morbidity of RP [3]. The impact of the surgery on men’s well-being is well documented. The quality of life (Qol) of those who underwent radical prostatectomy is significantly worse when compared with their noncancer counterparts [4]. Among several domains of Qol, the urinary function has been extensively investigated, including urinary incontinence as one of the most bothersome lower urinary tract symptoms (LUTS). Although less frequently studied, other LUTS are not uncommon after RP [5]. Unfortunately, the high preoperative incidence of LUTS further complicates research on the impact of surgery on lower urinary tract (LUT) function afterwards [6,7,8,9].

Conditions that cause postprostatectomy urinary incontinence are well known [10,11,12], yet the number of other bladder function alterations that may influence the postsurgical urinary domain of quality of life (QOL) have not been thoroughly investigated. Among others, these apply to detrusor underactivity, detrusor overactivity, bladder outlet obstruction, and reduced bladder compliance. Although novel tools are evolving [13], the most precise method to evaluate them all is the urodynamic pressure-flow study (UDS). Therefore, the aim of our systematic review is to evaluate the impact of radical prostatectomy on bladder function, with special attention towards detrusor underactivity investigated with the means of urodynamic evaluation.

## 2. Materials and Methods

The conduct and reporting of this systematic review were performed in accordance with the guidelines of the Centre of Reviews and Dissemination as well as the Preferred Reporting Items for Systematic Reviews and Meta-Analyses (PRISMA) statement [14,15]. The review protocol was registered in the PROSPERO International prospective register of systematic reviews (ID#: CRD42020223480). The studied population was limited to men with prostate cancer who underwent urodynamic study both prior to and at least once after radical prostatectomy. Studies without preplanned UDS before and after RP were excluded in accordance with the review protocol. Inclusion and exclusion criteria are summarised in Table 1.

The main outcomes appraised in this study are the incidence of detrusor underactivity (DU) that occurred after RP (de novo) as well as the total rate of DU in patients after RP. Additionally, the incidence of de novo and total detrusor overactivity (DO), impaired bladder compliance (IBC), and bladder outlet obstruction (BOO) after RP were assessed.

**Figure 1:**
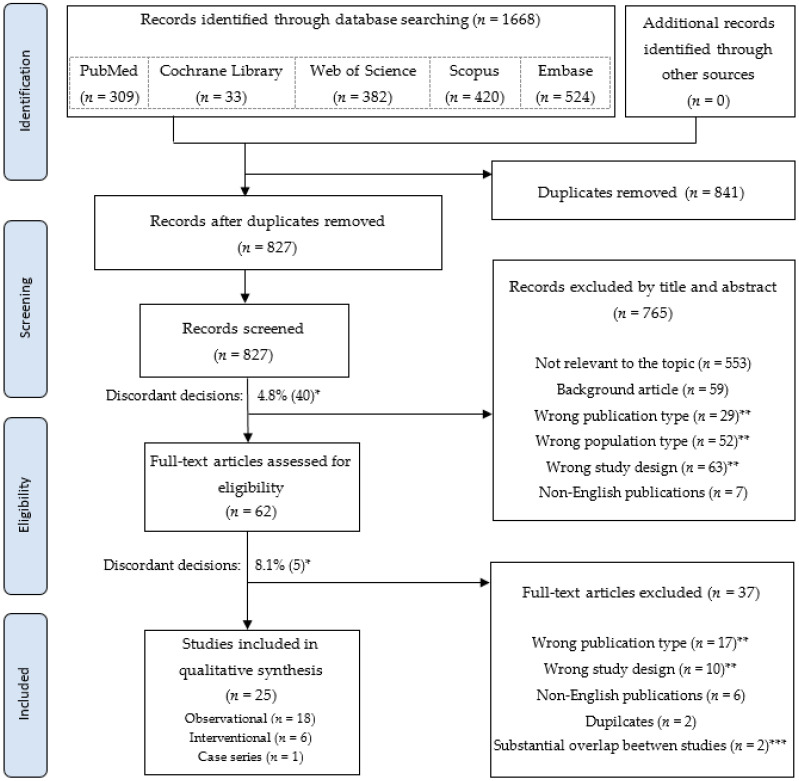
PRISMA diagram. The flow of records through the searching and appraisal process. * percent (number) of discordant decisions between investigators, resolved by consensus. ** according to the criteria presented in Table 1. *** based on additional data obtained from the author.

Electronic literature searches were conducted on 10 November 2020 using Pubmed, Cochrane Library, Web of Science, Scopus, and Embase databases. The search was then rerun on 10 December 2021, prior to the final analysis. The staged process of studies selection is illustrated in Figure 1, while detailed searching strategies for each database are listed in Supplementary Materials (Table S1). Screening, assessment against the predetermined eligibility criteria, and risk of bias assessments (National Heart, Lung, and Blood Institute Study Quality Assessment Tools) were performed by two researchers independently (M.O., K.B.). An online, cloud-based tool (http://rayyan.qcri.org, accessed on 12 November 2020) was used for the screening and eligibility assessment process [16]. Researchers were blinded to each other’s results, whereas discordant decisions were resolved by consensus with the assistance of an additional independent researcher (M.K.). Data were collected into electronic data extraction form, which was first piloted and then applied by two independent investigators (M.O., K.B.). Collected data includes study characteristics, demographic data, characteristics of the intervention, baseline and follow-up urodynamic data, and patient-reported outcome measures (PROMs).

## 3. Results

A total of 827 studies were screened, 62 identified as relevant and 27 initially qualified for synthesis [17,18,19,20,21,22,23,24,25,26,27,28,29,30,31,32,33,34,35,36,37,38,39,40,41,42,43] (Figure 1). Populations from three [26,27,28] of the last-mentioned studies were, however, substantially overlapped (72% to 90%, based on data obtained from the author), which led to further exclusions [27,28]. Finally, 25 studies were included in the qualitative synthesis, with 16 of them being prospective studies. Four of included twenty-five articles came from two authors, indicating some risk of population overlap [19,20,32,33], despite differences in years of inclusion, number of patients and populations characteristic. Attempt to contact authors for clarification was ineffective in these cases.

Included articles vary substantially in terms of length of follow up, type of intervention, and definitions of reported outcomes. Considering this heterogeneity, quantitative synthesis was not performed.

### 3.1. Baseline Characteristics

The total number of patients in included studies was 1387 (age range 13–110). In the case of 47 patients, no adequate data was reported [24,30,31]. Finally, the number of patients included in qualitative synthesis was 1340 (age range 13–110) (data presented in Table 2), with a mean age range from 61.9 to 72.1 years (SD 2.6). At least one UDS after RP was performed in 1298 from 1340 patients (97%). The total of 42 from 1340 patients had no UDS postoperatively (Table S2) [22,25,35,36,38]. Patients in thirteen studies were treated with open radical prostatectomy (ORP) [17,18,19,20,21,25,30,31,35,37,38,39,41]. Two studies reported outcomes after laparoscopic radical prostatectomy (LRP) [22,32] whereas five studies after robot-assisted laparoscopic radical prostatectomy (RALP) [23,26,40,42,43]. Four studies included a mixed population of patients treated either with ORP or LRP [24,33,34,36]. In one study, either LRP or RALP was performed [24]. Application of nerve-sparing technique was reported in 15 studies, ranging from 0% to 100% in treated patients [33,40]. The presence and range of lymphadenectomy performed during surgery were not reported in the majority of articles. Urodynamic evaluation in most reports adheres to rules outlined in the Good Urodynamic Practice statement [44]. Baseline demographics of included studies are collected in Table 2.

### 3.2. Postoperative Reporting

The length of follow-up ranged from 1 to 36 months [39,42], with the first assessment being performed from 3–4 days following catheter removal to 12 months after surgery [29,42]. Status of urinary continence was the most often reported functional outcome after RP (in 19 studies), although based on different timespans and assessment tools. Incontinence rates ranged from 0% to 98%.

#### 3.2.1. Detrusor Contractility

Detrusor contractility was assessed in 10 studies embracing in total 603 patients (Table 2) [18,19,20,26,29,32,34,35,36,42]. Definitions of detrusor underactivity (DU) and mathematical tools used for its assessment varied, however, substantially between studies (Table 3). Rates of impaired detrusor contractility before surgery were reported in eight studies with a median of 40.8% (1.6% to 75%) [18,42]. In one study, baseline detrusor contractility was not evaluated at all [32], whereas in another, bladder contractility was characterised as a continuous variable [29]. Absolute rates of DU initially after RP increased in five studies [19,20,26,35,36] with range from 13.4% to 42%, however in only two, these changes were significant (18.4% and 20.5%, *p* < 0.01) [19,20]. Furthermore, both reports came from the same author, indicating some risk of patients overlap. In three of the five last-mentioned studies, rates of DU insignificantly decreased afterwards with a range from 11.1% to 34.3% [19,20,26]. In the other two studies, DU was assessed only once after surgery (6.5 and 12 months, respectively), and no significant changes of DU were revealed [18,34]. In one study, bladder contractility increased in the term of contractility pattern [29].

Rates of de novo DU after RP were reported in eight studies [19,20,26,32,34,35,36,42] with a median of 29.1% (9.1% to 37%) on the first follow-up. In the case of three of those studies, further follow-up assessment was conducted, revealing de novo DU in 6% to 25% of evaluated patients. On the other hand, preexisting DU resolved in 7% to 35.5% of the studied population. PROMs were reported in four [29,34,36,42] of the ten studies assessing DU, with IPSS and its QOL domain being most often used (Table 3). Only in the study by Natsume et al. [36] the mean IPSS number significantly increased, whereas, in others, differences were insignificant.

The coincidence of DU and other urodynamic alterations was not uncommon (Table 3) and was reported in five studies [19,20,26,32,35]. The concomitance of DU and ISD (Intrinsic Sphincter Deficiency) was found to be the most frequent combination (range from 6% to 60%). The second most common one was the mixture of DU and DO, with a median of 29.6% (6% to 59%).

#### 3.2.2. Detrusor Overactivity

Detrusor overactivity was the most frequently reported outcome, being assessed in 23 studies (Table 2). The median rate of DO, preoperatively, was 25% (5% to 76%) in 18 studies [17,18,19,20,21,23,24,26,29,30,31,32,34,35,36,38,39,43]. The rate of de novo DO initially after RP was evaluated in 14 [18,19,20,23,24,26,29,30,31,32,34,35,36,39] studies, with the timing of the first follow-up ranging from a few days following catheter removal to 22 months. The median rate of de novo was 15% (2.3% to 54.4%). On the other hand, in ten studies [17,20,23,24,26,30,31,32,34,38], DO resolved initially in 19.6% to 87.5% of affected patients, with a median of 33%. During further observation in four studies [19,20,26,39], lasting from 6 to 36 months, DO occurred de novo in 1.4% to 15.9% (median of 9.5%) of patients and resolved in 25% to 42.8% of them (median of 25.9%).

Changes in absolute rates of DO on first follow-up evaluation differ substantially between 18 studies with a median of −2,5% (−29.6% to 56.2%) [17,18,19,20,21,23,24,26,29,30,31,32,34,35,37,38,39,43]. Rates of DO increased initially in seven studies with a median of 8% (6.4% to 56.2%) [19,20,23,31,33,35,39], but decreased in nine studies (median of 5.7%, range from 2.7% to 29%,) [17,18,24,26,29,30,34,38,43].

#### 3.2.3. Bladder Outlet Obstruction

Bladder outlet resistance was described preoperatively solely as a continuous variable (bladder outlet obstruction index—BOOI) in three studies [24,36,43] and as a rate of BOO in eight studies [18,19,20,26,29,31,34,39] (Table 2). Baseline rates of BOO varied between studies with a median of 27.8% (19% to 59.3%). The most pronounced change after surgery was a decrease of absolute rates of BOO, with a median of 19.8% (4.7% to 51.9%) and resolution of BOO in 88% to 93,8% (median of 92%) of affected patients (value relative to preoperative status) [20,26,31]. In minority of patients (median of 12%, range from 3.2% to 15.9%) BOO occurred de novo indicating usually formation of vesicourethral anastomosis stricture [19,20,26,31,35,36].

#### 3.2.4. Bladder Compliance

Bladder compliance was reported in 15 studies preoperatively [18,19,20,21,22,24,25,26,31,33,34,35,36,39,43] and in 12 [18,19,20,21,22,24,25,26,31,33,34,36,43] studies postoperatively (Table 2). In eight studies it was expressed as continues variable (mean compliance; ml/cmH2O) whereas in six studies it was stated as rate of impaired contractility (IBC, compliance < 20 mL/cmH_2_O) or both. In four studies rates of IBC increased initially ranging with median of 17.5% (6% to 40%) [18,19,20,26]. There was no initial decrease in rates of IBC reported, however it was reported during further observation (up to 36 months) in three studies with median of 25.6% (8.1% to 31.8%) [19,20,26]. Rates of the novo IBC were reported in four studies with median of 13.3% (3.2% to 41.3%) [18,19,20,26], whereas resolve of IBC was reported with median of 4.8% (0% to 47%) [19,20,26,34].

## 4. Discussion

This systematic review revealed a high incidence of bladder dysfunction in men qualified for RP. The median rates of DU, DO, and BOO preoperatively were as high as 25%, 40.8%, and 27.8%, respectively. Additionally, RP substantially impacted LUT, which was expressed by both resolution and de novo occurrence of selected alterations in bladder function. All studies report an almost entirely homogenous decline in absolute rates of obstruction after RP. A much more complex picture pertains to DU and DO. De novo DU was reported with a high divergence of rates which varied from 9.1% to 37% across studies. On the other hand, preexisting DU resolved in 7% to 35.5% of studied populations. Similar changes, although with greater intensity, were revealed in terms of DO. Resolution of preoperative DO was reported with rates from 19.6% to 87.5% (median of 33%). Additionally, in a substantial percentage of patients (2.3% to 54.4% with a median of 15%), the occurrence of de novo DO was found. Data corresponding to the influence of these alterations on patients’ complaints and quality of life is, however, inconsistently reported across studies.

Men newly diagnosed with organ-confined PCa are often affected by LUTS. Although the preoperative incidence of LUTS was not a point of preplanned synthesis in this review, it may be indirectly illustrated by rates of LUT dysfunction on urodynamic study preoperatively. The coexistence of PCa and LUTS may be explained by the fact that incidences of both increase with age [45,46], along with the phenomenon that patients with LUTS are more likely to be tested for PCa [47]. It has been previously shown that 12.1–56% of men undergoing radical prostatectomy (RP) have preoperative LUTS [48,49]. In a recent study by Walker et al., mild, moderate, and severe LUTS were reported by 50.7%, 39.4%, and 9.9% of patients, respectively [6]. This may be attributed to bladder dysfunction as well as BOO from benign or, less frequently, malignant enlargement of the prostate [6,50]. Detrusor dysfunction such as DO and DU were reported in 17.4% and 14.8% of men, respectively, whereas BOO in 29.5% [6]. This systematic review revealed higher median rates of these alterations, which may be due to the selection of screened population limited to patients who had UDS both before and after the surgery, according to the predefined outcomes. There is no doubt that high rates of preoperative DU, DO, and BOO complicate reliable assessment of the impact on LUT function RP may have. Therefore, it seems reasonable to operate with the rates of de novo (or the resolution of) DU, DO, and BOO after RP as surrogates of surgical influence on bladder function.

A wide range of reported rates of de novo DU and DO may be partially explained by divergent definitions of distinctive alterations across studies. It was particularly pronounced in the case of DU, with six different formulas used in 10 studies (Table 3). Furthermore, none of these were validated in the population of men subjected to RP [51,52,53,54]. The authors believe that the optimal method to evaluate bladder contractility in men after RP—the population with usually extraordinarily low outflow resistance—is the assessment of maximum isometric detrusor pressure [55,56,57]. Another reason for the variety of reported rates may be the disparities in the time between surgery and the first urodynamic assessment, which range from 10 days to 12 months. Considering the ability of the lower urinary tract to restore its function after RP, this may be an important factor [58]. De novo DU after RP was reported in 8 of 10 studies assessing bladder contractility. This indicates the relevancy of mentioned phenomenon. It is believed to be related to autonomic nerve damage during surgical dissection [19,20]. This applies specifically to the dissection in the proximity of the bladder neck and the removal of the seminal vesicles [59,60,61,62]. Contrary, reinnervation may explain the restoration of detrusor function in time [58], which was also reported in reviewed studies [19,20,26,29]. Furthermore, nerve-sparing surgery may lead to preservation of some autonomic nerves too, whereas pelvic lymphadenectomy, performed in selected cases, may escalate pelvic plexus injury. Although the status of nerve-sparing was reported in some of the reviewed studies, correlation with the rate of DU was not assessed. Only Hata et al. performed a multivariate analysis of factors that may contribute to the DU. Preoperative IBC was the only measure established as a predicting factor for the development of postoperative DU [42]. In regards to postulated pathophysiology, many different nerve-sparing techniques have been created to improve functional outcomes [63]. Despite differences, they aim in reducing injury to neurovascular bundles due to respect of anatomical details and avoidance of traction. In a recently published study by Cochetti et al., a novel RALP technique meeting these goals was validated as safe and effective with good functional outcomes [3].

Detrusor underactivity is widely known to cause a number of severe clinical problems, including voiding difficulties, retention, and urinary tract infections [64,65]. However, its relevance in patients with substantially decreased outflow resistance after RP is under debate with contradictory results regarding symptoms and QOL (Table 3). In the previously mentioned study by Hata et al., better results in terms of IPPS total score and QOL were observed in men without DU when compared with those with de novo DU, one month after the surgery [42].

A much more homogeneous influence of RP on BOO was reported, with all reviewed studies suggesting improvement in absolute rates of obstruction. As mostly attributed to BPH in this population, BOO is resolved by removing the prostate during surgery. Furthermore, some improvement is observed [29,34,36,39,48,49] in terms of LUTS, being likely related to BOO in older men [66]. This is claimed to be responsible for the net improvement in PROMs in some studies [36,39]. However, as in the noncancer population [67,68], in the studied cohort, not all LUTS could be attributed to preoperative BOO. The persistence of DO was reported in up to 51.5% [19]. This almost mirrors the data of BPH patients in whom DO resolution ranged from 57.1% to 83.3% after benign prostate surgery [69]. Changes after RP are, however, more complicated, with de novo DO ranging from 2.3% to 54.4% with a median of 15%. This result could be biased by the wide range of time span of postoperative assessment and heterogeneity of baseline populations across analyzed studies. Furthermore, it could be explained by an underestimation of BOO postoperatively.

A wide range of changes in the rate of DO after RP may also be explained by the inclusion of the study by Muccardi et al., which showed the highest rise in the postoperative rate of DO (by 56.2%) and the highest rate of de novo DO (54.4%), with no resolution of it in previously affected patients [35]. After exclusion of this paper, the range of de novo DO in remaining studies is from 2.3% to 20.9%, and the range of absolute changes in DO rate is from −29.6% to 12%. Outcomes regarding DO reported by Muccardi et al. may be explained by the high rate of vesicourethral anastomosis stricture (VAUS) (12%). Although those patients were excluded from postoperative UDS, these outcomes may raise a concern about subclinical VAUS, which may contribute to DO.

De novo DO is generally attributed to the partial denervation and devascularization due to the bladder neck mobilization, as well as alteration of bladder geometry after the surgery [20,39].

To the best of our knowledge, only a few systematic reviews have been published so far. The one conducted by Porena et al. included 19 studies, with only eight embracing postoperative and preoperative urodynamic findings [5]. Moreover, it was published in 2007, and therefore it did not include 20 relevant studies published up to date. At the time of conducting this systematic review, another review concentrating on the related problem was published [70]. The specific focus of our research is the detrusor underactivity, whereas Yao et al. concern all alterations equally. Additionally, we were able to include 10 relevant studies [17,20,23,25,32,34,40,41,42,43] missed by the other review. On the contrary, we omitted four studies included in the study of Yao et al.; two [27,28] were excluded due to the substantial overlap with the third study [26], one as it contains patients after salvage radiotherapy [71], and one because of the lack of postoperative UDS report [72]. Despite qualitative and quantitative differences in included studies between the reviews, conclusions are corroborated by the two.

Although being the most up-to-date and comprehensive summary of RP impact on bladder function, this systematic review has some limitations. Above all, it is limited by the level of evidence and the heterogeneity of included studies. This applies to different approaches to the intervention (RP), range of follow-up, as well as definitions and tools used to assess alterations of bladder function. There is also a lack of consensus regarding DU definition in this particular group of patients. Moreover, there are no randomised studies assessing the impact of RP on bladder function. It would be, however, very difficult to conduct such a study. Finally, some of the studies that fulfilled eligibility criteria suffer from the lack of adequate outcomes reporting. Authors postulate the need for further evaluation of bladder function alterations after RP in prospective studies with both validated PROMs and UDS. Considering the transient character of many functional alterations after prostate surgery and the time needed for recovery, the optimal period for such evaluation seems to be no sooner than 6 to 12 months after RP [58,73,74]. Furthermore, choosing proper tools for the assessment of bladder contractility after RP is challenging since outlet resistance is considerably low after surgery. Evaluation of isovolumetric bladder contraction with a stop test seems to be the optimal method for this purpose. Moreover, the potential influence of nerve-sparing and lymphadenectomy during RP on bladder function should be assessed.

## 5. Conclusions

BOO, DO, and DU are frequently diagnosed in men qualified for RP. BOO is improved after RP in the majority of patients; however, there is still a substantial rate of patients with persistent or de novo DU as well as DO. Hence, the impact of RP on the lower urinary tract seems to be more complex than that resulting from just BOO improvement. Both DU and DO may impair functional outcomes [75] and the quality of life; however, there is no adequate data, especially including PROMs, to make a final statement. Further studies are thus required to define factors that may predict the risk of permanent LUT dysfunction after RP and the potential role of UDS in that process.

## Figures and Tables

**Table 1:** Eligibility criteria.

Inclusion Criteria	Exclusion Criteria
(1) Studies on patients who underwent radical prostatectomy for the treatment of prostate cancer (open, laparoscopic or robotic). (2) Studies on patients who underwent urodynamic studies performed both before and after radical prostatectomy. (3) Full text published studies; articles in English only.	(1) Studies on patients who underwent radiotherapy for prostate cancer prior to surgery or during follow-up. (2) Studies on a predefined subgroup of patients undergoing radical prostatectomy, such as patients with neurogenic bladder, determined lower urinary tract symptoms, persistent postprostatectomy stress urinary incontinence.

**Table 2:** Qualitative synthesis of data from included studies.

Year	Author	N	F-UP (Months) (Range)	Age (Years) Mean (Range)	Type of RP	NS	UI	PVR (mL) ∆ PVR	Bcap. (mL) ∆ Bcap. (mL)	BC (mL/cmH_2_O) or/and IBC ∆ BC; ∆ IBC	BOO ∆ BOO	DO ∆ DO	DU ∆ DU
1984	Rudy	17	Pre-op.	64.2	ORP	-	19% (3/16)	-	365	-	-	25% (4/16)	-
		14	6				87% (14/16)	-	293 ↓ 72 †	-	-	14.3% (2/14) ↓ 10.7% **R 50% (2/4)**	-
1986	Hellstrom	19	Pre-op.	63 (49–75)	ORP	-	5.3% (1/19)	30 ± 10.3	550 ± 34.3	37 ± 10.40	-	5.3% (1/19)	-
		19	6				32% (16/19)	0 ± 4.68 ↓ 30 *	450 ± 35.1 ↓ 100 †	23 ± 3.16 ↓ 14 *	-	5.3% (1/19)	-
1992	Constantinou	13		62 ±1.7	ORP	61.5% (8/13)	-	150 ± 37	494 ± 42	-	-	76.9% (10/13)	-
		13	22.9 § ± 1.1	62 ±1.7			38.5% (5/13)	62 ± 43 ↓ 88 ± 32†	469 ± 55 ↓ 25	-	-	61.5% (8/13) ↓ 15.4% **N 8% (1/13)** **R 23% (3/10)**	-
1995	Connoly	17	Pre-op.	-	ORP	-	0%	54.3	441.9 ^a^	-	-	-	-
		17	Post-op.				0%	21.5	366.8 ^a^ ↓ 75.1 †	-	-	**N 11.8% (2/17)**	-
1999	Kleinhouse	44	Pre-op.	68	ORP	-	0%	38.1 ± 90.6	375.4 ± 171.9	-	-	36.4% (16/44)	-
		44	7.8 § (3–10)				15.9% (7/44)	5.4 ± 10.6 ↓ 32.7 †	427.8 ± 144.7 ↑ 52.4 *	-	-	6.8% (3/44) ↓ 29.6% **N 2.3% 1/44** **R 87.5% (14/16)**	-
2000	John	39	Pre-op.	-	ORP	20.5% (8/39)	0%	-	483 ± 168	49 ± 35	-	-	-
		34	1.5				82.4% (28/34)	-	376.8 ^a^	18.71 ^a^	-	-	-
		34	6				17.6% (6/34)	-	430.7 ^a^ ↓ 53.9	21.18 ^a^ ↑ 2.5	-	-	-
2004	Giannantoni	49	Pre-op.	65.3 ± 5	ORP	81.6% (40/49)	14.3% (7/49)	-	-	37.1 ± 14.9 20.4% (10/49) ^b^	57.1% (28/49)	55.1% (27/49)	42.8% (21/49)
		49	1				98% (48/49)	-	-	26.9 ± 12.7 38.7% (19/49) ^b^ ↑ 18.3% **N 18.4% (9/49)**	8.2% (4/49) ↓ 48.9% **N 8.2% (4/49)**	67.3% (33/39) ↑ 2.2% **N 12.2% (6/49)**	61.2% (30/49) ↑ 18.4% * N 28.6% (14/49) **R 23.8% (5/21)**
		49	8				59.1% (29/49)	-	-	29.2 ± 12.8 30.6% (15/49) ^b^ ↓ 8.1% **N 10.2% (5/49)**	0% ↓ 8.2%	65.3% (32/49) ↓ 2% **N 10.2% (5/49)**	42.8% (21/49) ↓ 18.4% * N 10.2% (5/49) **R 30% (9/30)**
2004	Natsume	17	Pre-op.	69.4 (62–76)	ORP/LRP	-	-	20 (0–103) 9.4 ± 14.8%	-	37.3 ± 28.1	22.9 ± 34.3 ^c^	17.6% (3/17)	35% (6/17)
		13	1				-	1.0 ± 2.5% ↓ 8.4%	-	16.1 ± 12.7 ↓ 21.2 *	5.0 ± 20.7 ^c^ ↓ 17.9	**N 15.4% (2/13)**	77% (10/13) ↑ 42%
		12	3				47% (8/17)	3.6 ± 8.9% ↑ 2.6%	-	32.8 ± 21.6 ↓ 4.5	18.8 ± 22.8 ^c^ ↓ 4.1 **N 15.4% (2/13)**	-	77% (10/13) **N 38% (5/13)** **R 10% (1/10)**
		12	6				29.4% (5/17)	4.0 ± 8.4% ↑ 0.4%	-	40.4 ± 31.5 ↑ 3.1	15.6 ± 35.2 ^c^ ↓ 7.3	-	-
2006	Majores	63	Pre-op.	61.9 ± 6.24	ORP	9.5% (6/63)	-	15.1 ± 9.2	337 ± 81.9	27.1 ± 7.3	19% (12/63)	23.8% (15/63)	-
		63	2				31.7% (20/63)	3.6 ± 14.9 ↓ 11.5 *	302 ± 52.2 ↓ 35 *	24.2 ± 7.2 ↓ 2.9 †	14.3% (9/63) ↓ 4.7% **R 92% (11/12)**	30.2% (19/63) ↑ 6.4% **N 15.9% (10/63)** **R 40% (6/15)**	-
2006	Noguchi	45	Pre-op.	68 ± 4.8	ORP	-	-	-	381.3 ^a^	-	-	-	-
		45	0.25				50.9% ^a^	-	240.9 ^a^ ↓ 140.4	-	-	15.3% ^a^	-
		45	1				33.2% ^a^	-	283.6 ^a^ ↓ 97.7	-	-	7.5% ^a^ ↓ 7.8%	-
		45	3				19.9% ^a^	-	326.1 ^a^ ↓ 55.2	-	-	5.3% ^a^ ↓ 2.2%	-
2008	Giannantoni	54	Pre-op.	67 ± 5	ORP	81.5% (44/54)	0%	-	N/A	38.3 ± 12 37.1%(20/54) ^b^	59.3% (32/54)	61.2% (33/54)	38.8% (21/54)
		54	8				70% (40/54) *	-	-	24.2 ± 9.9 ↓ 14.1 † 53.7% (29/54) ^b^ ↑ 20.6% * N 20.4% (11/54) **R 10% (2/20)**	7.4% (4/54) ↓ 51.9% * N 7.4% (4/54) **R 93.8% (30/32)**	70% (38/54) ↑ 8.8% † **N 14.8% (8/54)** **R 9.1% (3/33)**	59.3% (32/54) ↑ 20.5% * N 29.6% (16/54) **R 23.8% (5/21)**
		32	36				59.3% (19/32)	-	-	27.9 ± 11.4 ↑ 3.7 28.1% (9/32) ^b^ ↓ 25.6% **N 15.6% (5/32)** **R 25% (8/32) ^f^**	0% (0/32) ↓ 7.4%	56.3% (18/32) ↓ 13.7% **N 15.6% (5/32)** **R 25% (8/32) ^f^**	25% (8/32) ↓ 34.3% **N 25% (8/32)** **R 15.6% (5/32) ^f^**
2009	Matsukawa	63	Pre-op.	66.5 (53–75)	LRP	19% (12/63)	-	-	253.6 (64.8)	63.9 (31.8)	-	-	-
		63	4.3 § (3–9)				17.5% (11/63)	-	240.5 (60.9)	32.8 (14.8) ↓ 31.1 *	-	12.3% ^a^	-
		58	Pre-op.	67 (55–73)	ORP	0%	-	-	-	-	-	-	-
		58	6.1§(3–12)				22.4% (13/58)	-	206 ^a^	21.48	-	42.6% ^a^	-
2010	Matsukawa	110	Pre-op.	66.1 ^a^	LRP	-	-	-	255 ^a^	-	-	25.5% (28/110)	-
		110	3.8 § (2–5)				-	-	248.7 ^a^	-	-	32.7% (36/110) ↑ 7.2% **N 20.9% (23/110)** **R 53.6%(15/28)**	N 9.1% (10/110) *
2010	Song	72	Pre-op.	64 (49–77)	ORP	-	-	14.5 ± 18.2%	393 ± 91.5	2.8%(2/72) ^b^	20.8% (15/72)	38% (27/72)	4.2% (3/72)
		72	3				46% (33/72)	-	-	-	5.6% (4/72) ↓ 15.2%	45.8% ↑ 7.8% **N 16.7% (12/72)**	-
		72	6				18% (13/72)	-	-	-	2.8% (2/72) (↓ )2.8%	**N 1.4% (1/72)**	-
		72	36				-	-	322.3 ± 103.9	-	2.8% (2/72)	51.4% ↑ 5.6% **N 5.6% (4/72)** **R 25.9% (7/27)**	-
2012	Mitsui	43	Pre-op.	65 ^a^	ORP/LRP	-	0%	60.2 ± 86.3	379 ± 148	0%^b^	30.2% (13/43)	11.6% (5/43)	48.8% (21/43)
		43	12				-	22.7 ± 65.8 ↓ 37.5 *	320 ± 112	0%^b^	7% (3/43) ↓ 23.2% *	9.3% (4/43) ↓ 2.3% **N 9.3%(4/43)** **R 60% (3/5)**	44.2% (19/43) ↓ 4.6% † **N 9.3% (4/43)** **R 28.6% (6/21)**
2012	Dubelman	66	Pre-op.	64 (60–67) ‡	ORP	56.1% (37/66)	-		473	12% (8/65) ^b^	49.2% (31/63)	26% (17/66)	75% (48/64)
		66	6.5				28.9% (19/66)		435 ↓ 38 †	18% (12/65) ^b^	28.6% (18/63) ↓ 20.6% *	21% (14/66) ↓ 5%	70.3% (45/64) ↓ 4.7% †
2013	Mucciardi	100	Pre-op.	65.6 ± 5.4 (50–77)	ORP	-	-	-	-	16% (16/100) ^b^	-	12% (12/100)	73% (73/100)
		88	12			-	-	-	-	-	**N 9.3% (4/43)**	68.2% (60/88) ↑ 56.2% **N 54.5% (48/88)**	86.4%; (76/88) ↑ 13.4% **N 31.8%(28/88)**
2015	Kadono	63	Pre-op.	65.3 ± 4.8	RALP	58.7% (37/63)	0%		335.9 ± 92.3	28.3 ± 18.3 33% (21/63) ^b^	25.4% (16/63)	28.6% (18/63)	22% (14/63)
		63	Post-op.				84.1% (53/63)	-	251.4 ± 69.8	16.3 ± 10.8 ↓ 12 * 73% (46/63) ^b^ ↑ 40% * N 41.3% (26/63) **R 4.8%(1/21)**	6.3% (4/63) ↓ 19.1 **N 3.2% (2/63)** **R 88% (14/16)**	22.2% (14/63) ↓ 6.4 **N 3.2% (2/63)** **R 33.3% (6/18)**	49.2% (31/63) ↑ 27.2% **N 28.6% (18/63)** **R 7.1% (1/14)**
		63	12				11% (7/63)	-	338.1 ± 91.5	27.1 ± 21.7 ↓ 10.8 * 41.2% (26/63) ^b^ ↓ 31.8% * N 3.2% (2/63) **R 47.8% (22/46)**	3.2% (2/63) ↓ 3.1% **N 15.9% (1/63)** **R 75% (3/4)**	22.2% (14/63) **N 9.5% (6/63)** **R 33.3% (6/18)**	38.1% (24/63) ↓ 11.1% **N 6.3% (4/63)** **R 35.5% (11/31)**
2017	Jiang	46	Pre-op.	69.2 ± 7.9	LRP/RALP	34.8% (23/66)	-	31.6 ± 60.8	304.0 ± 131.7	68.1 ± 73.6	25.2 ± 33.7	56.5% (26/46)	-
		46	3–6				-	↓ 9.8 ± 60.0	-	↑ 6.6 ± 108.3	↓ 23.9 ± 37.4 *^,c^	52.2% (24/46) ↓ 4.3% **N 15.2% (7/46)** **R 19.6% (9/46)**	-
		46	12				-	↓ 5.2 ± 29.5	-	↓ 37.5 ± 112.9	↓ 34.1 ± 40.0 *^,c^	52.2% (24/46)	-
2017	Kitta	37	Pre-op.	65 (53–74)	ORP/LRP	46% (17/37)	-	48.6 ± 66.1	388 ± 139	-	24,2%(9/37)	10.8% (4/37)	114.6 ± 35.6 ^d^; −0.4 ± 2.0 ^e^
		37	12				-	10.1 ± 28.5 ↓ 38.5 *	351 ± 111	-	8.1% (3/37) ↓ 16.1%	8.1% (3/37) ↓ 2.7%	115.4 ± 18.2 ^d^; −2.2 ± 2.8 *^,e^
2018	Huang	48	Pre-op.	72.1 ± 5.68	LRP	37.5% (18/48)	-	31.3 ± 63.8	296.0 ± 106.9	65.3 ± 75.9	-	-	-
		31	1				93.8% (45/48)	30.26 ^a^	302.1 ^a^	65.4 ^a^ ↑ 0.1	-	-	-
		28	3				66.7% (32/48)	-	-	-	-	-	-
2019	Iguchi	75	Pre-op.	67.6 ^a^	RALP	44% (33/75)	0%	-	226.9 ^a^	-	-	25.3% (19/75)	-
		75	3				33.3% (25/48)	-	207.8 ^a^	-	-	33.3% (25/75) ↑ 8% **N 17.3% (13/75)** **R 36.8% (7/19)**	-
2020	Zhou	35	Pre-op.	63.4 ± 8.1	RALP	100% (35/35)	0%	-	385.3 (351.3–410.2)	-	-	-	-
		35	6				0%	-	370.2 (330.1–395.4)	-	-	-	-
2021	Hata	64	Pre-op.	66.1 ± 4.7	RALP	25% (16/64)	0%	-	-	-	-	-	1.6% (1/64)
		64	1				-	-	-	-	-	-	**N 37% 24/64**
2021	Lee	61	Pre-op.	69.0 (61.0–73.0)	RALP	82% (50/61)	-	41.0 (17.0–70.0)	306.0 (248.0 356.0)	57.0 (44.0–80.0)	36.0 (28.5–53.3)^c^	9.8% (6/61)	
		61	4				18% (11/61)	22.5 (10.0–56.0) ↓ 18.5 †	287.5 (229.3–340.8)	57.5 (41.0–80.0) ↑ 0.5 †	28.5 (15.0–40.0) ^c^ ↓ 7.5 *	5.0% (3/61) ↓ 4.8% *	-

Abbreviations: N = number of patients included; Pre-op. = preoperative; Post-op. = Postoperative; ORP = open RP; LRP laparoscopic RP; RALP = robot assisted laparoscopic RP; NS = nerve sparing surgery; UI = urinary incontinence; PVR = post-void residual; Bcap. = bladder capacity; BC = bladder compliance; IBC = impaired bladder compliance, % (n/N); BOO = bladder outlet obstruction, % (n/N); DO = detrusor overactivity, % (n/N); DU = detrusor underactivity, % (n/N). **∆** = indicate change in listed parameter (e.g., change in rate of BOO); If not indicated otherwise states for absolute change. Statistical significance or lack of significancy was indicated always when available. **Bold text** refers to rates of (**N**) de novo IBC, BOO, OD, DU or rates of IBC, BOO, OD, DU resolved **(R)** after RP. If not indicated otherwise **R** states for relative change. **N and R** are relative to previous follow-up assessment. § = mean; ‡ = median; * = *p* < 0.05; † = not significant; ^a^ = weighted arithmetic mean; ^b^ = % of patients with IBC; ^c^ = BOOI (bladder outlet obstruction index), mean (range); ^d^ = BCI (bladder contractility index); ^e^ = W80–W20 (W/m^2^), value of W at a relative volume rV = 0.80 minus its value at a relative volume rV = 0.20; ^f^ = in this case **R** relates to all patients available for follow-up.

**Table 3:** Characteristics of studies reporting DU.

Author	F-UP (Months) (Range)	Criteria of DU	Coincidence	PROMs
Giannantoni	Pre-op.		DU + BOO 16.3% (8/49) DU + DO 14.3% (7/49)	-
	1	Schafer nomogram	DU + DO 36.7% (18/49); ↑ 22.3% * DU + ISD 57.1% (28/49) *	-
	8		DU + DO 22.4% (11/49) † ‡ DU + ISD 30.6% (15/49) * ‡	-
Natsume	Pre-op.		-	IPSS 10.8 ± 8.3 (0–28); QOLsc 3.3 ± 1.9 (0–6)
	1	Schafer nomogram	-	IPSS 13.3 ±10.4; ↑ 3,5 *; QOLsc 4.3± 1.5; ↑ 1 *
	3		-	IPSS 9.5 ± 9.5; QOLsc 2.9± 2.1
	6		-	IPSS 6.1 ± 6.4; QOLsc 1.8± 1.3*
Giannantoni	Pre-op.		DO + DU 16.7% (9/54)	Strain voiders 20.4% 11/54
	8	Schafer nomogram	DO + DU 38.9% (21/54); ↑ 22,2% * N 29.6% (16/54) DU + ISD 44.4%(24/54) *	-
	36		DO + DU 21.8% (7/32) **N 12.5% (4/32)** DU + ISD 34.4(11/32) *	-
Matsukawa	Pre-op.		-	-
	3.8 § (2–5)	↑ Pabd and PdetQmax < 10 cmH_2_O	DO + de novo DU 0%	-
Mitsui	Pre-op.		-	IPSS 8.6 ± 7.0; QOLsc 3.4 ± 1.5 **DU** (post-op) **non-DU:** IPSS 10.4 ± 8.3 vs. 7.1 ± 5.5 † QOLsc 3.9 ± 1.5 vs. 2.9 ± 1.4 *
	12	WFmax < 10 (W/m^2^)	-	IPSS 7.9 ± 4.7†; QOLsc 2.5 ± 1.6; ↑ 0.9% * DU vs. non-DU: IPSS 8.6 ± 4.2 vs. 7.2 ± 5.2 † QOLsc 2.7 ± 1.4 vs. 2.2 ± 1.8 †
Dubelman	Pre-op.		-	-
	6.5	WFmax ≤ 10 (W/m^2)^	-	-
Mucciardi	Pre-op.		DU + DO 6% (6/100) DO + DU + IBC 6% (6/100)	-
	12	BCI < 75, WFmax < 7 μW/mm^2^ and MVDC < 7.5 mm/s.	DU + DO 59.1% (52/88); ↑ 53.1%; **N 52.3% (46/8)** DO + DU + IBC 25% (22/88); ↑ 19% **N 18.2% (16/88)**	-
Kadono	Pre-op.		ISD + DU 0%; DO + DU 0% DO + BOO 16% (10/63)	-
	Post-op.	Schafer nomogram	ISD + DU 60%; (38/63); ↑ 60%; **N 60% (38/63)** DO + DU 6% (4/63); ↑ 6%; **N 60% (4/63)**	-
	12		ISD + DU 6% (4/63); ↓ 54%; **N 0%** DO + DU 5% (3/63); ↓ 1%; **N 0%** DO + BOO 2% (1/63); **N 0%**	-
Kitta	Pre-op.		-	IPSS 8.8 ± 7.3; QOLsc score 3.5 ± 1.5
	12	BCI; rV (Wmax), Line(W) and W80–W20	-	IPSS 8.1 ± 4.8; ↓ 0.8† QOLsc score 2.5 ± 1.7; ↓ 1 *
Hata	Pre-op.		-	**Non-DU vs. DU** (post-op) IPSS 7.3 ± 5.7 vs. 11.7 ± 8.3 * QOL 2.6 ± 1.5 vs. 3.7 ± 1.5 * OABSS 2.8 ±2.5 vs. 3.6 ±2.0 †
	1	pdetQmax ≤ 25 cmH_2_O and Qmax ≤ 15 mL/s	-	**Non-DU vs. DU** (post-op) IPSS 11.3 ± 6.9 vs. 16.7 ± 9.0 * QOL 3.5 ± 1.9 vs. 4.4 ± 1.6 * OABSS 6.8 ± 3.9 vs. 9.2 ± 3.7 *

Abbreviations: N = number of patients included; Pre-op = preoperative; Post-op = Postoperative; DU—detrusor underactivity, ISD = intrinsic sphincter deficiency; PROMs = patient-reported outcome measures BCI = bladder contractility index; MVDC = maximum velocity of detrusorial contraction; WFmax = power at maximum flow; QOL = quality of life score; OABSS = Overactive Bladder Symptom Score. * *p* < 0,05; † = not significant; ‡ = compared to baseline; **N** = de novo, relative to previous follow-up assessment.

## Data Availability

All the data are available from the corresponding author upon reasonable request.

## Supplementary Materials

The following supporting information can be downloaded at: https://www.mdpi.com/article/10.3390/medicina58030381/s1, Table S1: Searching strategies for electronic databases; Table S2: Baseline characteristics of patients without follow-up.

## Abbreviations

IBC	impaired bladder compliance,
BOO	bladder outlet obstruction,
DO	detrusor overactivity,
DU	detrusor underactivity.
