# Supplementary material for: De Novo Detrusor Underactivity and Other Urodynamic Findings after Radical Prostatectomy: A Systematic Review
Source: Medicina (Kaunas). 2022 Mar 4;58(3):381. doi: 10.3390/medicina58030381 (PMC8949898; doi:10.3390/medicina58030381)
Supplement: Supplementary file 1 [file medicina-58-00381-s001.zip › medicina-1569746-supplementary.pdf]

Supplementary Materials

Table S1. Searching strategies for electronic databases.

Database	Query String
PubMed	(urodynami*[Title/Abstract] OR "detrusor underactivity"[Title/Abstract] OR "Impaired detrusor contractility"[Title/Abstract] OR "detrusor hypocontractility"[Title/Abstract] OR urodynamics[MeSH Terms] OR "pressure-flow"[Title/Abstract]) AND (radical[Title/Abstract] AND prostatectomy [Title/Abstract])
Cochrane Library	#1 (urodynami*):ti,ab,kw OR ("detrusor underactivity"):ti,ab,kw OR ("impaired detrusor contractility"):ti,ab,kw OR ("detrusor hypocontractility"):ti,ab,kw OR ("pressure-flow"):ti,ab,kw<br>#2 MeSH descriptor: [Urodynamics] explode all trees<br>#3 (radical):ti,ab,kw AND (prostatectomy):ti,ab,kw<br>#4 (#1 or #2) and #3
Web of Science	(TS = (urodynami*) OR TS = ("detrusor underactivity") OR TS = ("Impaired detrusor contractility") OR TS = ("detrusor hypocontractility") OR TS = ("pressure-flow")) AND (TS = (radical) AND TS = (prostatectomy))
Scopus	(TITLE-ABS-KEY (urodynami*) OR TITLE-ABS-KEY ("detrusor underactivity") OR TITLE-ABS-KEY ("Impaired detrusor contractility") OR TITLE-ABS-KEY ("detrusor hypocontractility") OR TITLE-ABS-KEY ("pressure-flow")) AND (TITLE-ABS-KEY (radical) AND TITLE-ABS-KEY (prostatectomy))
Embase	((urodynami*):ti,ab,kw OR ("detrusor underactivity"):ti,ab,kw OR ("impaired detrusor contractility"):ti,ab,kw OR ("detrusor hypocontractility"):ti,ab,kw OR ("pressure-flow"):ti,ab,kw) AND ((radical):ti,ab,kw ,AND (prostatectomy):ti,ab,kw)

Table S2. Baseline characteristics of patients without follow-up.

Year	Author	N	Reason for Lack of Postoperative UDS	Preoperative Urodynamic Findings			
				IBC	BOO	DO	DU
1984	Rudy	4	death: 1; resignation: 3	-	-	-	-
2000	John	5	vesicourethral anastomosis stricture	-	-	-	-
2004	Natsume	4	-	-	-	-	-
2013	Mucciardi	12	vesicourethral anastomosis stricture	0%	-	0%	12 (100%)
2018	Huang	17	-	-	-	-	-
